# Lessons learned from family-centred models of treatment for children living with HIV: current approaches and future directions

**DOI:** 10.1186/1758-2652-13-S2-S3

**Published:** 2010-06-23

**Authors:** Sarah C Leeper, Brian T Montague, Jennifer F Friedman, Timothy P Flanigan

**Affiliations:** 1 Brown University Medical School, Providence, Rhode Island, USA

## Abstract

**Background:**

Despite strong global interest in family-centred HIV care models, no reviews exist that detail the current approaches to family-centred care and their impact on the health of children with HIV. A systematic review of family-centred HIV care programmes was conducted in order to describe both programme components and paediatric cohort characteristics.

**Methods:**

We searched online databases, including PubMed and the International AIDS Society abstract database, using systematic criteria. Data were extracted regarding programme setting, staffing, services available and enrolment methods, as well as cohort demographics and paediatric outcomes.

**Results:**

The search yielded 25 publications and abstracts describing 22 separate cohorts. These contained between 43 and 657 children, and varied widely in terms of staffing, services provided, enrolment methods and cohort demographics. Data on clinical outcomes was limited, but generally positive. Excellent adherence, retention in care, and low mortality and/or loss to follow up were documented.

**Conclusions:**

The family-centred model of care addresses many needs of infected patients and other household members. Major reported obstacles involved recruiting one or more types of family members into care, early diagnosis and treatment of infected children, preventing mortality during children's first six months of highly active antiretroviral therapy, and staffing and infrastructural limitations. Recommendations include: developing interventions to enrol hard-to-reach populations; identifying high-risk patients at treatment initiation and providing specialized care; and designing and implementing evidence-based care packages. Increased research on family-centred care, and better documentation of interventions and outcomes is also critical.

## Background

Highly active antiretroviral therapy (HAART) has now been available for more than 10 years, profoundly changing the way we think about HIV, turning victims into survivors. Reliably robust results have been documented repeatedly in high- and low-income settings, with adults and with children [1,2]. Despite its long-standing record of proven efficacy, this treatment remains inaccessible to most children born with HIV in many low- and middle-income countries today.

In the five countries with the highest adult HIV prevalence worldwide, HIV is the single leading cause of under-five mortality, responsible for 41% to 56% of deaths [3]. One thousand children were born with HIV every day in 2007, due in part to the fact that only about 45% of all HIV-positive women worldwide have access to prevention of mother to child transmission (PMTCT) programmes [4]. Less than half of the children born with HIV in Africa are expected to survive until their second birthday [5].

With early diagnosis and treatment, however, their outlook improves substantially. For example, the Children with HIV Early Antiretroviral Therapy trial recently demonstrated a 76% reduction in mortality for children born with HIV when HAART was started within the first 12 weeks of life [6]. Among infected children of all ages, HAART initiation can decrease hospital admissions, incidence of pneumonia, and diarrhoea, can bring about "significant immunological reconstitution" and, in the sub-Saharan African context, result in a probability of survival after one year of therapy of between 84% and 97% [1].

**Figure 1:**
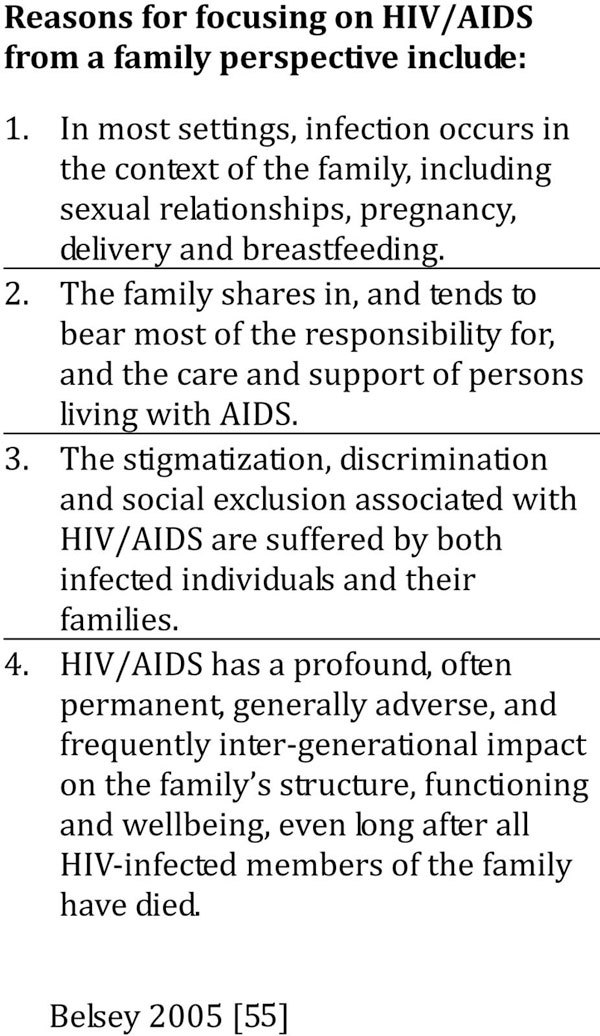
**HIV/AIDS from a family perspective**.

Children (infected and uninfected) also receive substantial indirect benefits when their parents are treated: decreases in malaria, diarrhoea, hospitalizations and mortality have been seen, as well as improvements in child nutritional status and school enrolment, and decreases in child labour [7-9]. In the context of HIV, family members have been shown to significantly impact the mental health, access to care, adherence and treatment outcomes of other family members [7,9-13]. However, only 38% of children and 43% of adults requiring antiretroviral therapy (ART) are currently able to access treatment [4]. Family-centred care models have emerged as a way to meet the clear and present need to test and treat more HIV-positive children and caregivers in a way that is mindful of intimate and dynamic family relationships (see Figure 1).

The concept of "family-centred care" was first formally defined in 1982 by the Association for the Care of Children's Health in response to a growing desire for a new approach to care for children with special health needs. It was based on a bio-psychosocial systems approach: the primary focus of health care is the client in the context of their family [14]. While the family was originally assumed to include healthy adults as caregivers for the child, definitions have evolved to meet the reality created through the vertical transmission of HIV. HIV family-centred care is now described simply as programmes where "adult and paediatric services are provided together in a single setting" [15].

While that is the working definition used in this paper, it is important to acknowledge that more ambitious definitions exist, which broaden the mandate of care providers beyond basic HIV services. For example, another definition is: "A comprehensive, coordinated care approach that addresses the needs of both adults and children in a family and attempts to meet their health and social care needs, either directly or indirectly through strategic partnerships and/or linkages and referrals with other service providers" [16].

There is currently no consensus as to what meeting "health and social care needs" means, as evidenced by the diversity of programmes reviewed in this paper. These myriad approaches illustrate the difficulty in drawing general conclusions about the efficacy of any given intervention, but also point to a broad global interest in exploring this care delivery model.

### Objectives

The goal of this paper is to review existing literature on family-care models used to treat children and caregivers living with HIV. The features of the HIV/AIDS family-centred care programmes, as well as paediatric cohort characteristics, are described, including demographics, treatment outcomes, adherence and retention. Lessons learned and recommendations for future interventions and research will be identified. Although the health of families is a complex and interrelated system, the focus will be mainly on the impact of the family-care model on the health of children living with HIV.

## Methods

The current study is a systematic review of English-language literature on family-centred HIV care programmes. Due to the low number of peer-reviewed publications on this topic, unpublished conference abstracts were also included. All relevant publication dating until August 2009 were identified by searching the PubMed database. The International AIDS Society (IAS) abstract search was used to identify abstracts, posters and presentations from the following conferences: 1^st ^to 5^th ^IAS Conferences on HIV Pathogenesis and Treatment (2001, 2003, 2005, 2007, 2009), and XIV to XVII International AIDS Conferences (2002, 2004, 2006, 2008).

The following search terms were used: ("famil*") + ("HIV" OR "AIDS" OR "HAART" OR "antiretroviral*"); also ("MTCT plus" OR "PMTCT plus"). Review of the citations within the articles found yielded additional articles. Final inclusion criteria included: (1) provision of treatment for HIV-positive adults and children in a single setting; and (2) a description of at least one of our measures of interest (services provided, cohort epidemiology, service uptake, testing, clinical/lab outcomes, adherence, retention, psychosocial support). Papers that did not address the treatment of HIV-positive children (such as publications on prevention of mother to child transmission or the follow up of HIV-exposed infants alone) were not included.

Data analysis primarily consisted of calculating ranges and measures of central tendency, when possible. Formal meta-analytic techniques could not be applied for a comparative analysis because of methodological and data collection discrepancies across studies.

## Results

Twenty-five publications and abstracts met inclusion criteria (cited throughout). Papers were published between 1997 and 2009, describing cohorts primarily in Africa, the US and the UK. Publications that were part of the Mother to Child Transmission Plus Initiative (MTCT-Plus) were considered separately if they were determined to describe discrete patient groups across unique time periods [17], while reports containing aggregate data on the same patient populations were not considered unique cohorts [18,19]. Similarly, results from two reports by Sendzik [20,21] detailing the Program for AIDS Treatment and Health (PATH) in Brooklyn, New York, USA, were combined.

Twenty-two separate cohorts were identified. All documented programme characteristics, and eight provided paediatric outcomes data [22-29]. See Additional File 1 and Table 1 for additional cohort references.

**Table 1:** Paediatric cohort characteristics and outcomes

Author/Date	# children on HAART	Age at initiation	Duration of follow up	CD4 at initiation	Adherence	Survival	Loss to Follow-Up
Abrams 2005 [18]	144		Median 19 months (Range 2 months - 12 years)				
Van Griensven 2008 [28]	332	Median 7.2 years (IQR 4.5-10.4)	Median 2.0 years (IQR 1.2-2.6)	Median 14% (IQR 9-18%)	49%: >95% adherence 46%: >80% adherence	98% survival at 12 months 8 deaths (2.6% mortality)	12 children (3.8%)
Eley 2004 [22]	80	Median 1.25 years (Range .003-12.0)			"Most": >85% adherence	7 deaths (8.8% mortality)	4 children (5%)
Habibu 2006 [23]*	52				>95% adherence		0 children
Lusiama 2004 [24]*	393	Median 7.5 (years) (IQR 4.3-10.5)	Median 21.9 months (IQR 7.5-25.9)	Median 12% (IQR 7-18%)		30 deaths (8% mortality)	44 children (9%)
Midturi 2008 [25]*	56	Mean 39.6 months	Mean 14.7 months		77.8% adherence	1.8% mortality	1.8%
Reddi 2007 [26]	151	Median 5.7 years (Range 0.3-15.4)	Median 8 months (IQR 3.5-13.5)	Median 7.4% (IQR 2.1-13.7%)	59.6%: no missed doses 29.8%: >95% adherence	90.9% survival at 12 months 13 deaths (8.6% mortality)	0 children
Tonwe-Gold 2009 [27]	43		Median 12 months (IQR 5.0-15.0)			2 deaths (4.9% mortality)	0 children
Van Winghem 2008 [29]	657	Median 5.5 years (IQR 3.2-8.7)	Median 1.36 years (IQR 0.6-2.2)			95.3% survival at 12 months 7 deaths (6.7% mortality)	67 children (10.2%)

* Indicates that this refers to a conference abstract, rather than a published journal article

Note: An empty table cell indicates none of that type of data were available in that publication

### Setting

Nineteen reports detailed the physical location where the patients were treated. A significant majority (n = 11) were located in ambulatory HIV clinics affiliated with various hospitals: community, teaching, public, and paediatric. Gibb *et al *report that this decision "had the advantage ... of being non-stigmatising (other paediatric outpatient clinics are held in parallel)" [30]. At Red Cross Children's Hospital in South Africa, the programme includes an inpatient consultation service, created to optimize the care of patients in the early stages of therapy who require hospitalization [22].

Five family-care programmes were based at government primary health centres. These locations were often conveniently located in settlements where families lived, and at the time of enrolment, were already offering a full range of primary care services for adults and children, including TB care. One drawback was that women who were tested at antenatal clinics and referred to these centres for care often failed to present for enrolment: in Kinshasa, Democratic Republic of the Congo, for example, only 27% of eligible women presented with their newborns [31].

Four family-care sites were located at antenatal or PMTCT clinics: three were hospital affiliated and one was community based. Although this facilitated maternal follow up, Tonwe-Gold theorized that the location "may have prevented a larger number of men from choosing to access the services provided" [27].

### Staffing

Most programmes were staffed by a core multidisciplenary team, including doctors, nurses, social workers and/or counsellors. Some included gynaecologists, child life specialists, and/or nutritionists. However, to navigate the challenges of trained health care worker shortages, several programmes took more innovative approaches to staffing.

Programmes that were part of MTCT-Plus, supported by the International Center for AIDS Care and Treatment, assembled and trained multidisciplinary teams at each site. Personnel were trained using a specific MTCT-Plus curriculum focusing on the team as a whole [17]. In a separate intervention in Nigeria, Habibu *et al *trained paediatricians to manage both children and adults for HIV-related conditions and prescribe ART, instead of training adult physicians to treat children. However, they caution, "Staff motivation can be impacted by the complexity of managing both children and adults and the multiple needs of the family" [23].

Project sites in Rwanda and Kenya implemented task shifting measures to varying degrees. In Kigali, Doctors Without Borders-supported clinics piloted "health center/nurse-based care". Nurses were trained to initiate and change antiretroviral (ARV) treatment, and perform routine follow up. They observed a gradual decrease in the need of physician time from one full-time physician per 1500 patients to one per 3000 patients as the programme matured. To avoid overloading the nurses, other tasks were taken over by "new or reinforced cadres in the health centers": receptionists, community support groups, and lab staff [28].

In Kenya, "rapid turnover of trained medical staff " was identified as a major challenge. Van Winghem *et al *propose training selected HIV-positive patients as peer educators and counsellors to take over those responsibilities from paid staff, as the volunteers "would be more likely to remain long-term with the program" [29].

### Programme components

Programmes vary widely in terms of services provided (see Additional File 1). Some offer only comprehensive HIV care to children and adults, and others provide supplementary services, such as primary care for all family members (HIV positive and HIV negative), TB screening and isoniazid prophylaxis, reproductive health services, nutritional supplementation, play therapy for children, and terminal care services. Locations of the programmes determined to some extent which services were offered: antenatal clinic-based programmes were better equipped to offer PMTCT services [32], and paediatric hospital-based programmes were well positioned to mobilize inpatient consult teams [22].

### Enrolment

Enrolment points varied widely, and included: antenatal clinics, PMTCT programmes, adult/adolescent HIV clinics, inpatient adult and paediatric wards, maternal and child health clinics, and subsequent use of "index patients" within the recruited cohort to identify HIV positive family members. Many sites relied on a combination of the above techniques. The enrolment method often influenced the inclusion or exclusion or various demographic groups within the treatment cohort.

#### 1. MTCT-Plus

A commonly used and well-documented strategy is MTCT-plus, a model of care that was developed from the MTCT-Plus Initiative [19]. Pregnant women are tested at antenatal or PMTCT clinics and, if HIV positive, referred to the family-care programme; they become the "index women". Upon enrolment, they are encouraged to bring children and male partners for testing and, if necessary, treatment and care. Although these programmes are extremely effective at recruiting HIV-positive women and supporting prevention of mother to child transmission, they have documented little success in recruiting HIV-positive children into care.

**Figure 2:**
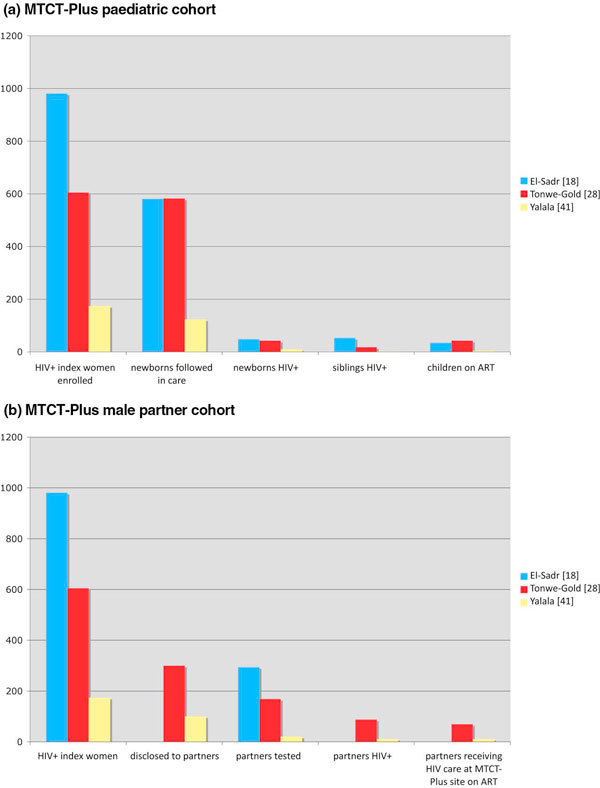
**(a) MTCT-Plus paediatric cohorts**. (b) MTCT-Plus male partner cohorts.

Figure 2 describes three MTCT-Plus cohorts: Tonwe-Gold in Cote d'Ivoire, Yalala in Kinshasa, Democratic Republic of Congo, and El-Sadr., which describes a composite cohort from 12 programmes in nine countries [17,27,31]. Despite a combined total of 1760 index women reported by the three authors, together they document only 74 children on HAART.

The uptake of testing for previously born children of the index women is particularly low. Various theories are offered, including "the possibility that many of the children lived away from the mother's household with other relatives in distant communities". This may be exacerbated by low rates of disclosure to a male partner, as revealing a child to be HIV positive might by extension reveal the mother's status. Figure 2b describes the same three cohorts in terms of partner enrolment. Again, Tonwe-Gold's study is the only one to document how many living male partners are reported (n = 568) [27].

#### 2. Other adult index patients

Other cohorts use adults (male and female) in their existing HAART cohorts as index patients to recruit other family members. Ninety percent of the children in a large Rwandan cohort were children of adult HIV positive patients (299 out of 332) [28]. Some programmes used incentives to encourage parents to enrol children: Sinikithemba Clinic in Durban, South Africa, offered free paediatric care to children whose parents were enrolled, and referred family members were prioritized for treatment [26]. Adult patients in Kenya were allowed to enroll in care at an earlier WHO clinical stage if they had a child in care [29].

Some cohorts have seen increased paediatric referrals since implementing family-centred care (the proportion of patients at Family AIDS Care and Education Services in Kenya who are children has doubled from 5% to 10%), but others are struggling to recruit paediatric patients [33]. At five health facilities in South Africa, HIV-positive patients were given referral cards to pass along to family members. Despite the fact that 33% of these adults reported not knowing their children's HIV status, the referred population was primarily adult (mean age 34 years) [34].

Only two programmes described interventions specifically aimed at increasing paediatric enrolment: developing a video for continuous playback in ART clinic waiting rooms encouraging parents in care to bring their children for testing [34]; and "in-depth counseling sessions ... with the caregivers to discuss testing of children in detail" [28]. Outcome data on these interventions are not currently available.

#### 3. Paediatric index patients

Some projects prioritize the recruitment of children, and rely on them to precipitate the diagnosis of adult family members. At Family Clinic for HIV at Tygerberg Academic Hospital in South Africa, the majority of infants and children living with HIV were identified through clinical suspicion based on hospitalization with "intercurrent disease or opportunistic infection". Parents were identified both through their children and with the input of the adult identification document service. However, the authors report "inadequate utilization by the parents, especially the fathers"; only 18% of potential parents attended the clinic [35].

In South London, UK, children were referred from a variety of sources, including paediatricians from district hospitals, social workers and general practitioners. The majority of parents had not been tested at the time their children first attended the HIV clinic, but in the five-year description of the programme, only 17% chose to remain untested. Again, the majority of adult patients who registered in care with their children were mothers (76%).

### Paediatric characteristics and outcomes

Paediatric baseline characteristics and outcomes were available for nine programmes. Very little data was available on clinical, immunological or virological outcomes. However, most studies documented cohort size, follow-up time, age of cohort, and rates of adherence, retention in care and mortality.

Cohorts contained between 43 and 657 children, and approximately one-third served <100. Median follow-up time after HAART initiation was recorded for eight cohorts, and ranged from 6.7 months to more than two years. Eight cohorts report average patient age at HAART initiation: half had a median age >5 years old, and half <5 years old, with two <2 years. CD4 percentage at initiation was reported by only three studies, and ranged from 7.4% to 14%.

Adherence data was available for six cohorts, and was assessed by methods ranging from patient self-report to pharmacy refill. The lowest adherence rate achieved was 77.8%, and four cohorts reported >95% adherence for the majority of their patients. Families on ART in Malawi, who are supervised for adherence by treatment helpers selected among HIV-positive clients, achieved an adherence rate of 99.7% [36]. Byakika-Tusiime *et al *note "near perfect adherence to ART" in both mothers and children when treatment was provided to all eligible HIV-positive family members [37].

In a particularly striking case study of a family with six family members living with HIV, all of whom were started together on HAART in rural Kenya, "excellent outcomes" were achieved despite a family total of 49 individual pill or syrup administrations daily [38]. These assessments, though imprecise, compare favourably to those of similar cohorts [2].

Excellent attendance at scheduled clinic visits was documented in several cohorts. The Global HIV/AIDS Initiative Nigeria Project in Kano, Nigeria, reports that in nearly a year of managing 202 children and 90 parents, only two clients missed scheduled clinic appointments [23]. In fact, family-care patients seem to be more likely to attend scheduled visits: in 2007, adults in the Family Program at PATH (the HIV service of Brooklyn Hospital, New York) kept 74% of their medical visits, compared to 44% for PATH patients overall [20,21].

Loss-to-follow-up (LTF) rates were low in the majority of studies: 10 report <11% LTF, including Ida *et al*, who demonstrated >90% retention during a seven-year observation period. Three cohorts report zero patients lost to follow up. One study, by Niekerk *et al*, reports 52% LTF, although this should be interpreted in light of the fact that this was predominantly a pre-HAART era report, and only 22% of the children were receiving HAART through various clinical trials [35].

The probability of survival one year after HAART initiation was 90.9% to 98% [26,28,29], and overall mortality ranged from 1.8% to 8.8% [22,24,25,27]. Several studies highlighted a particularly vulnerable period shortly after the initiation of HAART: all of the deaths (n = 7) reported by Eley took place within six weeks of HAART initiation, 70% of the deaths reported by Lusiama within three months, and all of the deaths (n = 13) reported by Reddi within five months [26,36,39]. This finding is consistent with the experience of other paediatric HIV treatment programmes in resource-limited settings [2].

Three articles identified predictors of mortality and LTF in family care cohorts. Reddi *et al *report that HIV-positive caregivers showed a protective effect against mortality when compared with caregivers who were untested or HIV negative [26]. Lusiama *et al *compared children in the family care cohort both with and without participating family members, and found that the rate of deactivation/death was higher among children without a family member participating in the programme [24]. A three-year retrospective case-control-matched study of children on ART enrolled at the Baylor Center of Excellence family clinic in Lilongwe, Malawi, and children receiving routine paediatric ART revealed better outcomes in family clinic cases compared with controls regarding retention in care, death, LTF, stopped ART, and transfer to other ART sites [25].

## Discussion

### Limitations

Due to the emerging and evolving nature of the family-centred care model, no fixed definition exists to facilitate the classification of programmes as family-centred or not. Consequently, studies included in this review were chosen on the basis of self-identification. Additionally, no consistency across studies exists with regard to programme components or data collection, precluding rigourous comparison and evaluation. Given the low number of peer-reviewed publications on this topic, a significant number of conference abstracts were also included in order to provide a more complete picture of the work being done "on the ground".

### Challenges to care and management - lessons learned

Preliminary data from family-centred care sites suggest that this model can be an effective tool for recruiting HIV-positive women, preventing mother to child transmission, increasing paediatric and adult referrals, supporting patient adherence and clinic attendance, and improving paediatric clinical outcomes. The data also describe a number of challenges encountered by programmes in their efforts to provide comprehensive health care for the whole family.

The majority of programmes described here reported challenges in recruiting one or more types of family members: females, males and children. Those with robust paediatric cohorts often struggled to recruit parents, and those with large numbers of HIV-positive mothers in care had great difficulty recruiting male partners and children. Fathers were the least likely to access care in all scenarios: as Tonwe-Gold wryly observed, involving males in family services like MTCT-Plus "is known to be very taxing" [27].

Failure of HIV-positive females to disclose their status to male partners has been well documented: fear of accusations of infidelity, abandonment, discrimination, loss of economic support, and violence are often cited as primary reasons. These fears are not groundless. A review of 17 studies found that between 3.5% and 14.6% of women reported experiencing a violent reaction from a partner following disclosure; other negative outcomes included separation from partner, abuse by in-laws, or being forced to move away from home [40]. Low levels of disclosure may negatively affect not only the likelihood that fathers will enrol in care, but also that mothers will seek testing and treatment for their children.

Several studies described the failure of the "trickledown" method of paediatric enrolment. The assumption that adults in care will refer their children for testing and treatment is not borne out by the clinical evidence and requires serious reconsideration.

Children living with extended family are made particularly vulnerable to exclusion from treatment. By 2010, it is estimated that 20 million children in sub-Saharan Africa - 12% of all children in the region - will have been orphaned by AIDS [41]. In Namibia, Tanzania and Zimbabwe, the United Nations Children's Fund (UNICEF) reports that grandmothers are responsible for the care of 40% to 60% of orphaned children. According to Mudzingwa and Reddi, non-parental caregivers are significantly less likely to know their own status, and thus to be in care for HIV [26,42]. Therefore, family-care models that depend solely on adult index patients are likely to miss the substantial proportion of HIV-positive children who live with non-biological caregivers.

DeGennaro suggests that family-centred programmes are able to "locate infections at earlier disease stages", and there is some tentative data to support their success in this endeavour [43]. Although age of enrolment is not an ideal surrogate for disease stage, it is the best indicator available, and there is likely to be some overlap between the two. Half of the family-centred HAART cohorts had a median paediatric cohort age of <5 years, whereas a review of paediatric antiretroviral cohorts in sub-Saharan Africa showed that only about 1/4 of their cohorts had a median age of <5 years [1].

MTCT-Plus programmes have documented particularly strong results: in Uganda, less than 1% of HIV-exposed infants in the programme died before testing [32]. Abrams *et al *reported that in 2004, a remarkable 37% of the paediatric cohort at all MTCT-Plus sites worldwide was less than one year of age [19]. However, it is necessary to find ways to replicate this success with infants who have a greater risk of infection, such as those whose mothers did not participate in MTCT.

The frequency of paediatric deaths at the onset of HAART, documented by Reddi, Eley and Lusiama, reflects a much larger trend across paediatric HIV treatment models. Sutcliffe, in a comprehensive review of paediatric HIV cohorts in sub-Saharan Africa, reports that "most deaths occurred within 6 months of treatment, with several studies reporting a mean or median time to death of 57-182 days" [2]. Identifying high-risk patients at the onset of treatment is an urgent necessity, especially in family-centred care settings where family members receiving treatment at the same site are well-positioned to serve as allies in the care of the high-risk child.

Finally, many of the programmes reviewed here have structural difficulties that limit their ability to provide comprehensive paediatric and adult care. A survey of non-governmental organizations by DeGennaro reveals "lack of healthcare workers trained in pediatrics" as the most common reason for the failure to provide treatment to children with HIV [44]. This sentiment is echoed in surveys of barriers to paediatric care in Zambia [45], South Africa [46] and district hospitals throughout Africa [47]. In Malawi, Lesotho, Swaziland and Botswana, per capita numbers of paediatricians range from 0.2 to 2.5 per 100,000 children [48].

Even trained staff can be overwhelmed by the increased volume of patients, or may view the attention to paediatric care in addition to adult care as an unnecessary burden. Finally, the simple logistics of finding space for additional programming at already overcrowded clinics may be difficult. In a community-based government health clinic in Kenya, "There was limited physical capacity of the clinics to provide child-specific activities and rooms" [29].

### Recommendations and interventions - a way forward

#### 1. Goal 1: Expand patient recruitment efforts

New methods of patient recruitment must be incorporated into family-centred care provision if more children are to be diagnosed, and diagnosed at earlier stages of illness. A variety of opportunities present themselves, including: immunizations, postpartum care, sick/well baby clinics, and inpatient paediatric wards. These sites would allow identification of both symptomatic and asymptomatic children, and include children with non-biological caregivers who might otherwise be missed in a parent-centred care and recruitment model. Studies addressing the acceptability of such interventions have found that routine HIV counselling and testing could be successfully incorporated into immunization clinics, paediatric inpatient wards, malnutrition treatment programmes and paediatric emergency departments with high parental acceptance rates [49-52].

It is also important to develop thoughtful, context-specific interventions both to support adult HAART patients' referral of their partners and children, and to encourage the caregivers of paediatric HAART patients to be tested themselves. These efforts need to take into account the very real danger faced by many women worldwide when disclosing their status to a partner. Counsellors should be trained to identify women most at risk for negative outcomes, and provide additional support, including referral to domestic violence services when necessary [40].

Interventions that might support the positive participation of males in HIV testing and treatment include utilizing male health care workers and counsellors, and establishing "fathers' clinics" or similar male-centred activities as an opportunity for education and peer support [53].

#### 2. Goal 2: Pay special attention to children during the first six months of HAART

While not specific to family-centred care, the unacceptably high risk of mortality for paediatric patients during the first six to 12 months of HAART needs to be addressed by all paediatric providers.

Integration of family-centred services may be useful in mitigating some of these risks. Incorporating therapeutic and supplementary feeding with HIV treatment programmes could support patients who are malnourished, and combining HIV care with TB screening and treatment might result in a lower TB incidence at baseline. Reddi *et al *recommend children identified as high risk at baseline be referred to paediatric inpatient wards or a local palliative (step-down) care centre for HAART initiation [26]. Other simple measures could include scheduling more frequent follow-up appointments after initiation, or treatment counsellor home visits. With the appropriate support, adult family members in care at the same treatment site could provide invaluable support and expertise during this treacherous time.

#### 3. Goal 3: Develop comprehensive services

At this point, it is difficult to identify which components of a family-centred care programme might be the most crucial and efficacious. Tolle, in advocating for a package of primary health services for comprehensive family-centred HIV/AIDS care, acknowledges that "implementing (packages) will require substantial and long-term investments in infrastructure and human resources". However, in the short term, services packages may present "a framework around which a programme may construct its own particular model of care, providing those services for which it is able while finding a reference point for the development of its future capacities" [15].

Additionally, establishing a consensus as to which interventions define family-centred care would allow researchers not only to independently validate discrete interventions, but also to compare broadly the effects of a standardized set of interventions comprising "family-centred care" versus more traditional segmented adult and paediatric care.

For these reasons, we suggest here, in Table 2, a "wish list" of services, compiled from the recommendations of Tolle, Richter, DeGennaro, and DeBaets [15,43,47,54].

**Table 2:** Family-centred care "wish list"

HIV + TB care	Paediatric + adult primary care	Psychosocial/economic support	Administrative
• PMTCT	• Immunizations	• Adherence counselling for adults and children	• Follow up and patient tracking
• VCT, including viral diagnostic tests for early infant diagnosis	• Growth monitoring	• Psychosocial support for both HIV+ and HIV- caregivers, including substance abuse, mental health, and domestic violence education	• A tight network of referrals and linkages with community-based organizations
• Opportunistic infection prophylaxis	• Routine neurodevelopmental assessments	• Psychosocial support for children: social and educational activities	• Monitoring and evaluation systems
• HAART for adults and children	• Nutritional supplementation and infant feeding support	• Early childhood development programmes	
• Regular TB screening, INH prophylaxis, and treatment	• Reproductive health services, including cervical screening and STD care	• Subsidized patient transport to and from the clinic	
	• Family planning services	• Income assistance	
	• Insecticide-treated bed nets, malaria screening and treatment		
	• Management of other endemic disease (e.g., helminths)		
	• Management of chronic illness: cardiovascular disease, Type II diabetes, hyperlipidemia		
	• Safe drinking water		
	• Pain management and palliative care		
	• Home health visits for pregnant mothers and young children		

Tolle, Richter, DeGennaro, and DeBaets [16,48,52,59]

## Conclusions

Family-centred care can be implemented in developed and developing world settings. Although data is currently limited, and additional research is urgently required, family-centred care produces good outcomes in terms of service uptake, clinical outcomes, adherence and retention.

Important considerations for future programming include building personnel and infrastructural capacity, innovating methods for testing hard-to-reach populations within the family, identifying and implementing specialized services for high-risk populations early in treatment, and providing a full range of comprehensive services for all family members. Additionally, more consistent documentation of programme experiences, and efforts to reach consensus around key definitions, would promote the development of understanding of how, and when, family-centred care is most effective.

## Competing interests

The authors declare that they have no competing interests.

## Authors' contributions

SCL undertook the study concept and design, and analysis and interpretation of data. BTM, JFF and TPF were responsible for critical revision of the manuscript for important intellectual content. All authors have read the final manuscript and approved it for publication.

## Supplementary Material

Additional file 1 **Family-centred care programme data**. Click here for file
