# Supplementary material for: Lessons learned from family-centred models of treatment for children living with HIV: current approaches and future directions
Source: J Int AIDS Soc. 2010 Jun 23;13(Suppl 2):S3. doi: 10.1186/1758-2652-13-S2-S3 (PMC2890972; doi:10.1186/1758-2652-13-S2-S3)
Supplement: Additional file 1 — Family-centred care programme data. [file 1758-2652-13-S2-S3-S1.doc]

­

| **Author/Date** | **Region** | **Cohort** | **Intake/referral** | **Location** | **Services** |
| --- | --- | --- | --- | --- | --- |
| Porricolo  2006 [12]* | Bronx, New York City, USA | 26 mother-child dyads |  | Hospital-based outpatient HIV clinic | Comprehensive HIV care, including HAART |
| Sendzik  2008 + 2006  [20, 21]* | Brooklyn, USA | 200+ clients | Clients may enrol if they have children at home, or are considering having children | Hospital-based HIV clinic | Comprehensive HIV care, including HAART  Primary care for HIV+ and HIV- family members  Ob/gyn nurse midwife  Family nutritionist  Family counsellors  Educational and social activities |
| El-Sadr  2004 [17]* | 12 programmes in 9 countries | 981 HIV+ index women  276 HIV+ partners  48 HIV+ index neonates  53 HIV+ children  36 children on HAART | MTCT-Plus |  | Comprehensive HIV care, including HAART  PMTCT |
| Van Griensven 2008 [28] | Kigali, Rwanda | 2937 adults on HAART  315 children on HAART | Paediatric cohort:  Children of HIV+ adult patients (90%)  Orphans (10%) 11  PMTCT and in/outpatient services (<1%)  Transfer from other ART programmes (<1%) | Community-based health centres | Primary health care  Comprehensive HIV care, including HAART |
| Callaway  1997 [39]* | Columbus, Ohio, USA |  |  | Children’s hospital-based outpatient clinic | Comprehensive HIV care, pre-HAART  Primary care for HIV+ and HIV- family members  Gynaecological care, including family planning  Substance abuse and mental health interventions  Nutritional assessment and support |
| Eley  2004 [22] | South Africa | 80 children on HAART  3 mothers on HAART |  | Children’s hospital-based HIV clinic | Comprehensive HIV care, including HAART  Inpatient consultation service |
| Gibb  1997 [30] | London, UK | 185 HIV+ children  112 HIV+ adults (24% fathers) | Children precipitate testing of parents | Children’s hospital-based HIV clinic | Comprehensive HIV care, pre-HAART  Terminal care services  Family counselling  Family planning service |
| Habibu  2006 [23] * | Kano, Nigeria | 52 children on HAART  22 parents started on ART. |  | Specialist hospital-based HIV clinic | Comprehensive HIV care, including HAART  Adherence nurse  Social worker |
| Himid  1998 [56] | South London, UK | 37 HIV+ mothers  47 HIV+ children | HIV+ mothers (pre-existing cohort) as index patients | Hospital-based outpatient clinic | Comprehensive HIV care, pre-HAART  Social workers  “Specialist health visitors” (paediatricians, midwives) |
| Ida  2006 [57]* | Brooklyn, USA | 47 families | The Obstetrics clinics where HIV-positive pregnant women were identified  Transfers from the Adolescent and Adult HIV clinic  Family members of index positive paediatric and adult patients |  | Comprehensive HIV care, including HAART  Full time adult providers & general paediatricians |
| Kabugo  2007 [32]* | Uganda | HIV+ index women (n = ?)  HIV+ male partners (n = ?)  HIV+ index neonates (n = ?)  HIV+ children (n = ?) | MTCT-Plus | Hospital-based antenatal clinic | Comprehensive HIV care, including HAART  PMTCT |
| Kiromera  2006 [36]* | Malawi | 2 infants on HAART  111 parents on HAART  (40% fathers) | MTCT-Plus | Hospital-initiated, community-based care | Comprehensive HIV care, including HAART  PMTCT  Education on appropriate infant feeding  Supplementary feeding to HIV-affected families Supervised for adherence by treatment helpers selected among HIV+ clients |
| Luisama  2008 [24]* | Kinshasa, Democratic Republic of Congo | 505 HIV+ children  393 children on HAART  Caregivers (n=?) |  | Paediatric hospital-based HIV clinic | Comprehensive HIV care, including HAART |
| Marima  2006 [33]* | Kenya | Children on HAART (n = ?)  Caregivers on HAART (n = ?) | Adult and paediatric patients refer each other |  | Comprehensive HIV care, including HAART  Referrals to community-based organizations for nutritional support, home-based care, and economic support |
| Midturi  2008 [25]* | Baylor College of Medicine, Lilongwe, Malawi | 56 HIV+ paediatric cases in family care  112 HIV+ paediatric controls in paediatric care |  |  |  |
| Okubamichael 2007 [58] * | Lesotho |  | MTCT-Plus | Hospital-based antenatal clinic | Comprehensive HIV care, including HAART  ART service at clinic run by medical officer 3x/week  PMTCT  Counselling on breastfeeding, postnatal/under-5 care |
| Reddi  2007 [26] | KwaZulu-Natal, South Africa | 151 HIV+ children  68 HIV+ caregivers | Adult and paediatric patients refer each other | Hospital-based outpatient clinic | Comprehensive HIV care, including HAART  Psychosocial support for adults and children |
| Tonwe-Gold  2009 [27] | Yopougon and Abobo (Abidjan, Cote d’Ivoire) | 605 HIV+ women  69 male partners  30 neonates (index pregnancy)  18 children | MTCT-Plus | Community-based antenatal clinics | Comprehensive HIV care, including HAART  Cotrimozaxole prophylaxis  Malaria treatment  Psychological and social support  Nutritional + infant feeding counselling and support,  Referral to family planning and tuberculosis services  Minimal fee for transport of patients to the clinic |
| van Kooten Niekerk  2006 [35] | Tygerberg, South Africa | 274 HIV+ children  97 parents (14% fathers) | “Clinical suspicion” in paediatric inpatients  The parents were identified through their children and with the input of the adult infectious diseases service | Hospital-based HIV clinic | Comprehensive HIV care, pre-HAART  Antiretroviral therapy was given to limited numbers of children and parents, either through pharmaceutical trials, medical insurance, donations, or through the hospital  Clinic operational on Tuesdays |
| Van Winghem 2008 [29] | Kenya | 1205 HIV+ children  657 children on HAART  HIV+ caregivers (n = ?) | Adult and paediatric HIV care cohort members as index patients – refer family members | 1 hospital-based HIV clinic +  3 community-based health centres | Comprehensive HIV care, including HAART  Primary care  TB care  Psychosocial support for adults  Psychosocial support for children  Nutritional support |
| Wamalume  2004 [59]* | Lusaka, Zambia | 60 index women  19 partners  34 children “enrolled”  (22 patients total on ART) | MTCT-plus  Women tested at ANC/postnatal clinics, referred to primary health centre for HIV care | Community-based health centre | Comprehensive HIV care, including HAART  INH prophylaxis against TB  Nutrition supplements from the World Food Programme. |
| Yalala  2008 [31]* | Kinshasa, Democratic Republic of Congo | 174 HIV+ index women  10 HIV+ index neonates  11 HIV+ male partners  2 HIV+ children | MTCT-Plus  Women tested at ANC, referred to primary health centre for HIV care | Community-based health centre | Comprehensive HIV care, including HAART  Primary care |

* Indicates that this refers to a conference abstract, rather than a published journal article

Note: An empty table cell indicates none of that type of data were available in that publication

[Please note: An empty box in the table indicates that the relevant data was not available in that
